# Nematode predatory ability of the fungus *Duddingtonia flagrans* affected by in vitro sequential exposure to ovine gastrointestinal tract

**DOI:** 10.1007/s11259-023-10089-y

**Published:** 2023-04-03

**Authors:** Elizabeth Céspedes-Gutiérrez, Diana Marcela Aragón, Martha Isabel Gómez-Álvarez, Jaime Andrés Cubides-Cárdenas, Diego Francisco Cortés-Rojas

**Affiliations:** 1 Corporación Colombiana de Investigación Agropecuaria AGROSAVIA, Sede Central, Mosquera, Colombia; 2 Universidad Nacional de Colombia., Sede Bogotá, Bogotá, Colombia; 3 Corporación Colombiana de Investigación Agropecuaria AGROSAVIA, Sede Tibaitatá, Mosquera, Colombia

**Keywords:** Anthelmintic resistance, Biocontrol, Gastrointestinal nematodes, Integrated pest management

## Abstract

*Duddingtonia flagrans* is a nematophagous fungus employed as a biocontrol agent of gastrointestinal nematodes in ruminants. After oral ingestion and passage through the digestive tract of animals, this microorganism captures the nematodes in the feces. The drastic conditions of ruminant digestive tract could affect fungi chlamydospores and therefore biocontrol activity. The aim of this study was to evaluate in vitro the effect of four ruminant digestive segments on the concentration and nematode predatory ability of a Colombian native strain of *D. flagrans*. The sequential four-step methodology proposed evaluated conditions of the oral cavity, rumen, abomasum, and small intestine such as pH (2, 6, 8), enzymes (pepsin, pancreatin), temperature (39 °C), and anaerobiosis comparing short (7 h) and long (51 h) exposure times. The results showed that the nematode predatory ability of the fungi is affected by sequential exposure to gastrointestinal segments and this effect depends on the exposure time to those conditions. After short exposure (7 h) through the four ruminant digestive segments, the fungi had a nematode predatory ability of 62%, in contrast, after long exposure (51 h) the nematode predatory ability was lost (0%). Moreover, the number of broken chlamydospores was higher in the long-exposure assay.

## Introduction

Drug-resistance to synthetic anthelmintics is a global problem related to the intensive use and inappropriate administration specially in small ruminants (Márquez 2003; Ploeger and Everts 2018; Castro Arnáez et al. 2021). The nematophagous fungi *Duddingtonia flagrans* is used for biological control of gastrointestinal nematodes in ruminants (Liu et al. 2020). Chlamydospores are orally administrated and are excreted in the feces where the fungi traps the nematodes (Ojeda-Robertos et al. 2009; Sagüés et al. 2011). *D. flagrans* chlamydospores are thick-walled with globular protuberances on the surface when they are mature (Wang et al. 2015). These structures protect the fungus against physical, chemical or microbiological stress conditions (Wang et al. 2019); however, the drastic environment of the ruminant digestive system reduce its biocontrol activity (Larsen et al. 1998; Clark 2004; Jiménez and Benítez 2005; Ojeda-Robertos et al. 2009). According to this, it is useful to know the effect of each segment of the gastrointestinal tract on the survival of chlamydospores, in order to design a delivery system that improves the viability of the fungi.

The survival of *D. flagrans* to gastrointestinal conditions has been evaluated in vivo and the final concentration and viability of the fungi in feces were correlated with the initial concentration orally administered (Ojeda-Robertos et al. 2008). Other studies have evaluated fungal survival making fistulas in the animal’s digestive tract (Larsen et al. 1998). These studies have some disadvantages such as the high cost related to the maintenance of the animals and the qualified personnel requirement for their handling. Additionally, from a practical point of view, its execution is laborious and time-consuming, which prevents the analysis of a large number of samples (Chandrawathani et al. 2003; Khan et al. 2015; Ortiz Pérez et al. 2017). Moreover, in vitro studies allow isolate the impact of each digestive segment.

In vitro methods that simulate gastrointestinal tract cavities are a valuable technique for quickly determining the effects to which the fungi will be exposed as it passes through the ruminal digestive system (Clark 2004). The methodologies employed are based on the procedure described by (Tilley and Terry (1963)(Tilley and Terry (1963)) to evaluate forage digestion which uses discontinuous containers incubated with ruminal fluid and a buffer, in anaerobic conditions at 39 °C (Martínez Peláez 2009). In these methodologies, only the ruminal and abomasal digestion were considered (Larsen et al. 1991; Clark 2004; Ojeda-Robertos et al. 2009; Victoria and Osorio 2013; Freitas et al. 2019). The aim of this study was to evaluate consecutively four gastrointestinal tract segments including the oral cavity and the small intestine in addition to ruminal and abomasal segments which are commonly evaluated. Critical response variables associated to fungi biocontrol activity were monitored: concentration and nematode predatory ability.

## Materials and methods

The study was carried out in AGROSAVIA (corporación colombiana de investigación agropecuaria) at bioproducts department located in Tibaitatá research center, Mosquera, Colombia.

### *Duddingtonia flagrans* biomass

The strain of *D. flagrans* coded as: BGMSABV-Df-Col-H-001–2014 was originally isolated from soil samples from the Cota region, Cundinamarca—Colombia (4.8099° N, 74.1018° W). The use of this microorganism was authorized by the access contract to genetic resources No. 168 of 2017. The fungus is preserved at -20 °C in a solution composed of glycerol 10% (w/w) and peptone 1% (w/w). The fungus biomass employed in each assay was produced by biphasic fermentation following the methodology described by Castillo-Saldarriaga et al. (2020). Concentration and nematode predatory ability were evaluated for each fungus fermentation batch (Céspedes Gutiérrez et al. 2021). A minimal concentration of 1 × 10^7^ chlamydospores/g and in vitro nematode predatory ability higher than 90% were established as acceptance parameters.

### Ovine rumen fluid

The ruminal fluid used in the in vitro methodology was obtained from a healthy 3-year-old wool sheep (Creole cross with Corriedale) fed with *Pennisetum clandestinum* grass with ruminal fistula according to the authorization of Agrosavia Ad Hoc Bioethics Committee (Act 033 of 2019). Ruminal fluid was extracted in the morning after 15 h of fasting, filtered (100 mesh), and placed in a tempered thermo flask to be used in the assays within 1 h. The pH of the rumen fluid was 6.69 ± 0.30.

### Evaluated variables

#### Chlamydospore concentration (chlamydospores/mL)

Chlamydospores were counted using a Neubauer chamber in a light microscope at 40 × magnification according to the methodology described by (Clark 2004). Chlamydospores with visual damage on structure were counted separately and results were expressed as a percentage of broken chlamydospores.

#### Nematode predatory ability (%)

The in vitro nematode predatory ability was evaluated according to the methodology proposed by (Mendoza de Gives 2011;) using the biological model of the nematode *Panagrellus redivivus* (Braga et al. 2012) and described by Céspedes Gutiérrez et al. (2021). The Baermann funnel technique was used to separate the larvae that were not captured by the fungi. The recovered larvae were counted, and the percentage of nematode predatory ability was calculated compared to a control (Rodríguez-Martínez et al. 2018).

#### In vitro methodology of the ovine gastro-intestinal tract

**Fig. 1:**
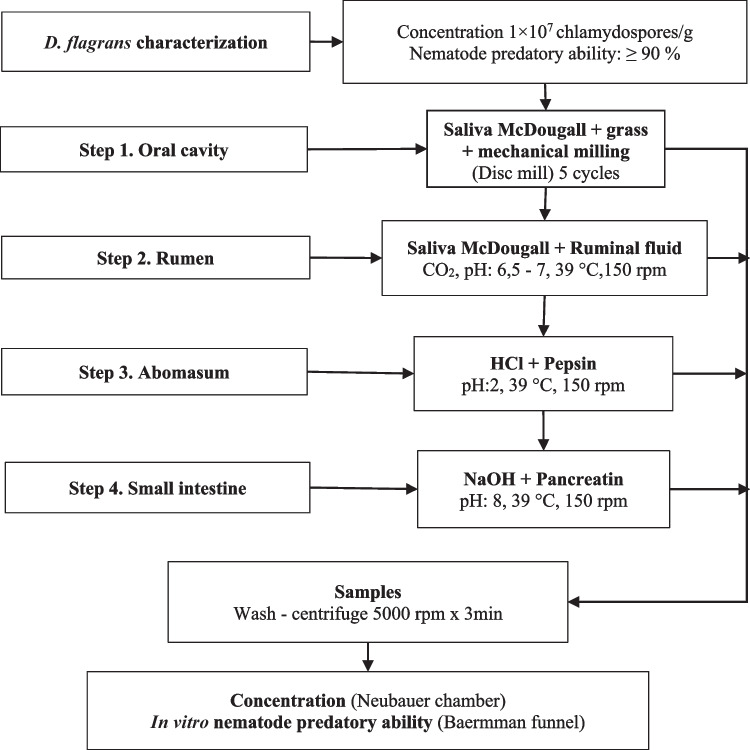
In vitro methodology to evaluate the effect of ovine gastrointestinal tract on the concentration and nematode predatory ability of *Duddingtonia flagrans*

The methodology proposed was developed considering previous experimental work, a literary review of in vitro digestion techniques, and the physiology of the gastrointestinal tract of ruminants. Considering the fluctuations in the duration of the digestion process related to factors such as feed composition, water intake or pathologic conditions, minimum and maximum exposure times were evaluated in each segment (Table 1) (Bines and Davey 1970; Thomas and Campling 1977; Dulphy et al. 1980; Vega et al. 1998). The proposed methodology consists of 4 sequential steps which are presented in Fig. 1 and described in detail below.

**Table 1:** Exposure times (hours) and milling cycles* (MC) of *Duddingtonia flagrans* to each segment of the ovine gastrointestinal tract

Gastrointestinal segment	Short exposure	Long exposure
Oral cavity	5 MC	5MC
Rumen	3 h	24 h
Abomasum	1 h	3 h
Small intestine	3 h	24 h
Total exposure time	7 h	51 h

^*^A milling cycle was defined as the total passage of the mixture through the disc mill.

- *Step 1. Oral cavity:* for the evaluation of this cavity the artificial chewing technique to predict the voluntary consumption of fodder in ruminants developed by Troelsen and Bigsby (1964) was used. The representation of this compartment started with the salivation and mixing of feed. Initially, 20 mL of artificial saliva ((McDougall 1947) was mixed with 6.4 g of *Pennisetum clandestinum* grass (dry and cut in short length fragments < 1 cm), then 40 g of biomass was added to the mixture, manually homogenized and mechanically ground by a disk mill (Landers & Cia) with a gap between the stationary and rotating disc of maximum 1 mm. Samples of 5 g were taken every 5 milling cycles. A cycle was defined as the total passage of the mixture through the disc mill. As a control treatment, the biomass was used without the milling process. Each sample was suspended in 50 mL of Tween® 80 0.1% w/v; subsequently, the concentration and nematode predatory ability of chlamydospores were evaluated. Additionally, the number of broken chlamydospores was counted and the result was expressed as a percentage.
- *Step 2. Rumen*: the evaluation of this segment was based on the in vitro technique of Tilley and Terry (1963). Initially, 8.3 g of the ground mixture obtained from the oral cavity (step 1) was weighed in triplicate to obtain a concentration of 1 × 10^6^ chlamydospores/g and placed in 3 Erlenmeyer of 250 mL. Consecutively, 80 mL of a mixture of ruminal fluid and McDougall's artificial saliva (1:4) was added and aerated with CO_2_ to achieve a pH in the range of 6.5 to 7.0 and immediately sealed with a rubber stopper provided with a gas regulation valve and submitted to constant agitation at 150 rpm at 39 ± 2 °C (Labtech ModelLSI-1005R). During the incubation time, the pH was monitored and adjusted (every 3 h) with NaOH 3N or HCl 0.5% solutions as required (pH 6.5–7.0 rumen treatment and pH 7.0 control treatment). At each sampling time, the temperature of 39 ± 2 °C (IKA® C-MAG HS7 heating plate) and the anaerobiosis (CO_2_) conditions were maintained in the flasks.
- *Step 3. Abomasum:* the evaluation of this segment was based on the in vitro digestibility technique reported by Tilley and Terry (1963) for the evaluation of forage and Ojeda-Robertos et al., (2009) for the evaluation of *D. flagrans* digestibility. At the end of the incubation time of the ruminal segment (step 2), the pH of the mixture of each Erlenmeyer was adjusted to 2.50 using a 50% HCl solution (1:1 HCl/water). Subsequently, a 50 mg/mL pepsin (0.7FIP-U/mg, Merck, 1.07185) stock solution in HCl 1N was added to a final concentration of 4.75 mg/mL. Erlenmeyers were sealed and submitted to agitation as described for the ruminal segment effect. At each sampling time, the pH was adjusted as required (2.0–2.5 abomasum treatment and 7.0 control treatment).
- *Step 4. Small intestine*: The methodology of this segment was based on the in vitro technique developed by Calsamiglia and Stern (1995) to estimate intestinal protein digestion in ruminants. At the end of the incubation time of the abomasum (step 4) the content of each Erlenmeyer was adjusted to pH 8.0 with a NaOH 6N solution. Consecutively, pancreatin (Sigma-Aldrich, P1750) was added to a final concentration of 3.38 mg/mL. During the incubation time, the pH was monitored and adjusted (every 3 h) as necessary (small intestine to 8.0 and control to 7.0).
  For ruminal, abomasal, and small intestine, the control consisted of three Erlenmeyer flasks with 5 g of biomass without any mechanical milling process in 80 mL of sterile water with 1.1 mL of a chloramphenicol solution (0.5 g/100 mL ethanol) under constant stirring at 150 rpm, 28 ± 2 °C and sealed with a rubber stopper provided with a gas regulation valve. At each sampling time an aliquot of 2 mL was taken per flask, centrifuged at 5000 rpm for 3 min (Hettich EBA 20 centrifuge) and 8 mL of sterile water was added to the resulting pellet. Subsequently, the pellet was resuspended in 2 mL of sterile water for nematode predatory ability assay.

#### Statistical analysis

Statistical analyses and graphics were completed employing the program GraphPad Prism (version 8.0.1). Data of the effect of the oral cavity in vitro were subjected to t-test comparison. The data of the effect of the rumen, abomasum, and small intestine were subjected to a two-way repeated measures ANOVA followed by Sidak´s post-hoc test at *p* < 0.05.

## Results

The first step of the in vitro methodology was the evaluation of the concentration and nematode predatory ability of *D. flagrans*. The batch used for the long-exposure assay had a concentration of 1.56 ± 0.57 × 10^7^ chlamydospores/g and a nematode predatory ability of 99.28 ± 0.88%. The batch used for the short-exposure assay had a concentration of 1.94 ± 0.54 × 10^7^ chlamydospores/g and a nematode predatory ability of 90.17 ± 4.04%.

After the first step of the methodology (oral cavity), the concentration of chlamydospores of the groups (control and treatment) was not significantly different for the short-exposure assay (t_6_ = 0.2156, *p* = 0.837), however, in the long-exposure assay the treated group was significantly different to its control t _6_ = 3.450, *p* = 0.014, (Table 2). After 5 cycles of mechanical milling, the concentration remained above 1.6 × 10^7^ chlamydospores/g. The number of broken chlamydospores for the short-exposure assay was 8.13 × 10^5^ chlamydospores/g, which represents 3.82% of the total chlamydospores and for the long exposure, it was 1.25 × 10^6^ chlamydospores/g, representing 3.28% of the total chlamydospores. In fact, the nematode predatory ability of *D. flagrans* did not present significant differences in the short-exposure assay compared to its control (t_14_ = 1.320, *p* = 0.208) it remained above 90%. In contrast, in the long-exposure assay, statistically significant differences were observed with the control (t_14_ = 2.612, *p* = 0.021), and the percentage of nematode predatory ability was greater than 97% (Table 2).

**Table 2:** Effect of the in vitro conditions of the oral cavity (5 cycles of mechanical milling) on the chlamydospores of the fungi *Duddingtonia flagrans*

Assay	Group	Log Concentration (chlamydospores/mL) Average (SD)	Nematode predatory ability (%) Average (SD)
Short exposure	Control	7.27 (0.14)^a^	90.17 (4.04)^a^
	Oral cavity	7.30 (0.19)^a^	92.17 (1.49)^a^
Long exposure	Control	7.17 (0.17)^a^	99.29 (0.88)^a^
	Oral cavity	7.56 (0.15)^b^	97.72 (1.44)^b^

SD: standard deviation (*n* = 3). The values with different superscript letters in columns indicate significant differences between groups (control and oral cavity) for each assay (short- and long exposure), according to t-test with a 95% confidence interval, *p* < 0.05.

**Fig. 2:**
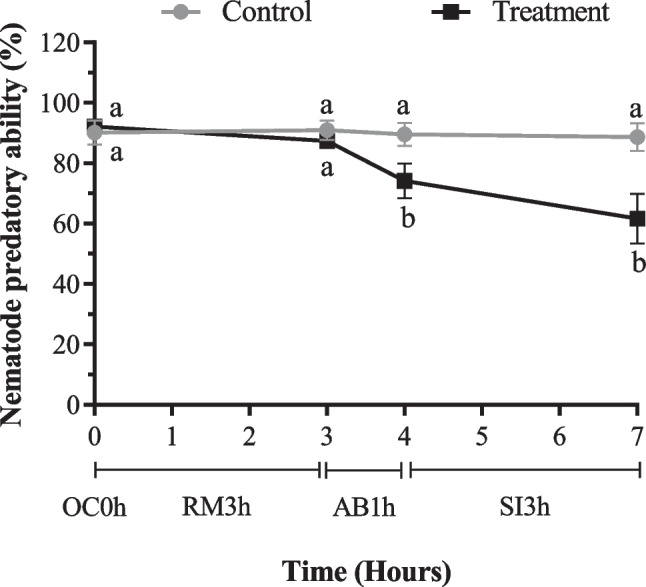
Effect of the short exposure in vitro methodology on the nematode predatory ability of the fungi *Duddingtonia flagrans*. Different letters indicate significant differences between groups (control or treatment) in each segment evaluation. OC0h: Oral cavity 0 h, RM3h: Rumen 3 h, AB1h: Abomasum 1 h, SI3h: Small intestine 3 h. Data are presented as mean and standard deviation (*n* = 8)

The sequential passage of *D. flagrans* through each gastrointestinal segment at the short-exposure assay had no significant effect on the concentration of chlamydospores (means are presented in Table 3) (*p* = 0.501, F _2, 30_ = 0.7149), however, the nematode predatory ability of the fungi (Fig. 2) was affected (*p* < 0.0001, F _3, 42_ = 97.48). By contrast, in the long-exposure assay, there was a significant effect on the two response variables (concentration *p* = 0.0009, F _2, 20_ = 10.27, nematode predatory ability of the fungi *p* < 0.0001, F _3, 42_ = 1594). The interaction of the exposure time with the groups (control or treated) had significant effects on the nematode predatory ability of the fungi (short exposure *p* < 0.0001, F _3, 42_ = 75.56, long exposure *p* < 0.0001, F _3, 42_ = 14.25) but not on the concentration (short exposure *p* = 0.1044, F _2, 20_ = 2.535, long exposure *p* = 0.5507, F _2, 20_ = 0.6146). The group (control or treated) had a significant effect on concentration (short exposure *p* = 0.0001, F _1, 10_ = 37.83, long exposure *p* = 0.0001, F _1, 10_ = 37.63) and nematode predatory ability of *D. flagrans*. (Short exposure *p* < 0.0001, F _1, 14_ = 33.01, long exposure *p* < 0.0001, F _1, 14_ = 33.46).

**Table 3:** Effect of in vitro methodology with short- and long-exposure times on the concentration of chlamydospores of the fungi *Duddingtonia flagrans*

Exposure time	Group	Log Concentration (chlamydospores/mL) Average (SD)		
		Rumen	Abomasum	Small intestine
Short		3 h	1 h	3 h
	Control	5.72(0.03) ^a►^	5.85(0.19) ^a►^	5.81(0.08) ^a►^
	Treatment	6.06(0.12) ^b►^	5.93(0.13) ^a►^	6.06(0.11) ^b►^
Long		24 h	3 h	24 h
	Control	5.83(0.15) ^a►^	5.93(0.12) ^a►^	5.99 (0.12) ^a►^
	Treatment	6.04(0.17) ^b●^	6.25(0.10) ^b►^	6.26(0.09) ^b►^

SD: Standard deviation, (*n* = 3). Values with different superscript letters in rows indicate significant differences between groups (control or treatment) and different superscript symbols (triangle or circle) in columns indicate differences between segments, according to Sidak´s test with 95% confidence interval, *p* < 0.05.

In the short and long-exposure assays the Sidak's multiple comparisons tests showed significant differences between the concentration of each evaluated cavity (rumen, abomasum, and small intestine) and its corresponding control (*p* < 0.05) (Table 3). In all cases the concentration of the treated group was higher than its control, however, in both groups, the concentration was higher than the expected value (1.0 × 10^6^ chlamydospores/mL). No significant differences were observed between cavities in the control group (*p* > 0.05) but in the treated group of the short-exposure assay differences were observed through each cavity (*p* > 0.05). In contrast, statistically significant differences (*p* < 0.05) were observed in the concentration of chlamydospores of the treated group at the long-exposure assay comparing the ruminal segment with the abomasum and small intestine (Table 3). In the control group, no significant differences were observed between cavities. Regarding the broken chlamydospores counts, no broken chlamydospores were detected in the controls of the two assays (short and long exposure). In the short-exposure assay, the concentration of broken chlamydospores in the treated group did not present significant differences (*p* > 0.05) between rumen (5.42 × 10^4^ chlamydospores/mL), abomasum (3.77 × 10^4^ chlamydospores/mL) and small intestine (6.95 × 10^4^ chlamydospores/mL). The percentage of broken chlamydospores compared to the total chlamydospores in the rumen is equivalent to 4.59%, in the abomasum to 4.25%, and in the small intestine to 5.92%. These results show an increase in the number of broken chlamydospores at the end of the short-exposure assay at the three cavities (7 h) with respect to the percentage of broken chlamydospores from the oral cavity (3.82%). On the other hand, with maximum exposure times, the concentration of broken chlamydospores in the rumen (6.0 × 10^4^ broken chlamydospores/mL) was not statistically different (*p* > 0.9999) from the abomasum (9.98 × 10^4^ broken chlamydospores/mL), but it was from the small intestine (1.28 × 10^5^ broken chlamydospores/mL) (*p* = 0.0039); in the same way, the small intestine was significantly different from abomasum (*p* = 0.0039). The results obtained indicate that the percentage of broken chlamydospores in the rumen was 5.14%, abomasum 5.54%, and the small intestine 6.87% considering the total number of chlamydospores. In all segments, the percentage of broken chlamydospores was higher than that obtained after the oral cavity exposure (3.28%). These results are higher compared with the minimum exposure times indicating that the sequential and prolonged passage through each segment affects the concentration and nematode predatory ability of chlamydospores.

**Fig. 3:**
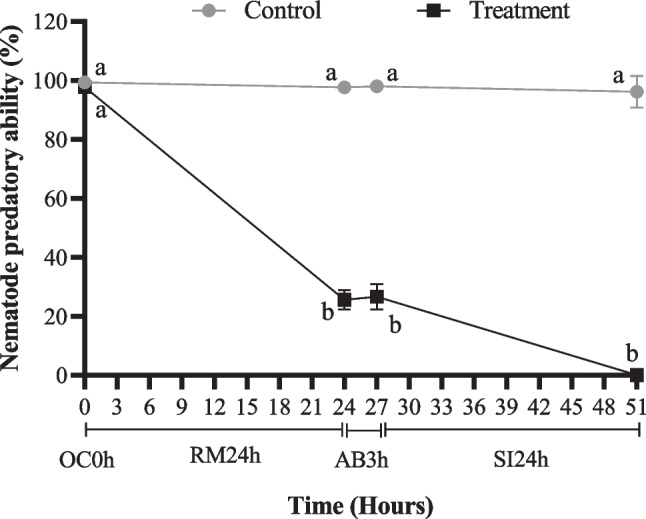
Effect of the long exposure to in vitro methodology on the nematode predatory ability of the fungi *D. flagrans*. Different letters indicate significant differences between groups (control or treatment) in each segment evaluate. OC0h: Oral cavity 0 h, RM24h: Rumen 24 h, AB3h: Abomasum 3 h, SI24h: Small intestine 24 h. Data are presented as mean and standard deviation (*n* = 8)

Figures 2 and 3 show the results of nematode predatory ability of the fungi *D. flagrans* after the short and long exposure to the in vitro methodology respectively. There were not statistically significant differences (*p* > 0.05) in the percentage of nematophagia in the control group during the long or short-exposure assay. The nematode predatory ability after short exposure was in a range of 91% to 88% and after long exposure in a range of 96% to 99%. The chlamydospores of the treated group with short exposure showed significant differences (*p* < 0.05) between the results obtained in the oral cavity (92%), rumen (87%) abomasum (74%), and small intestine (62%), moreover differences were observed between control and treated group in the abomasum and small intestine (*p* < 0.05) but not between oral cavity and rumen (*p* > 0.05)*.* In the long-exposure assay, the treated group presented a nematode predatory ability of 98% after the oral cavity, which is significantly higher (*p* < 0.05) than the rumen, abomasum, and small intestine, where drastic reductions were observed. The nematode predatory ability after the rumen for the treated group was 25.67% and it is not statistically different (*p* > 0.05) from the abomasum (26.60%). After the small intestine, the nematode predatory ability was 0% and therefore significantly different (*p* < 0.05) compared to the rumen and abomasum. Comparing the control group with the treated group in each gastrointestinal segment, significant differences were observed in the rumen, abomasum and small intestine but not in the oral cavity.

## Discussion

In the present work, the effect of an in vitro methodology representing segments of ruminal digestive tract was evaluated on the fungi *D. flagrans.* The methodology consisted of the sequential passage through the oral cavity, rumen, abomasum, and small intestine, with short and long exposure times to each digestive segment. The physicochemical, microbiological, and enzymatic conditions of the represented segments are challenging for the survival of the chlamydospores of the fungi which must be excreted in the feces to capture the nematodes. Two response variables were selected to assess the effect of the in vitro methodology, concentration, and nematode predatory ability, both have a direct relationship with the biocontrol activity of the fungi and could be altered by its exposition to adverse conditions.

Regarding the concentration of chlamydospores, the results showed that the exposition in vitro to the oral cavity, rumen, abomasum and small intestine do not cause a negative effect on the fungi since no significant reduction was observed, with neither of the two exposure times. Instead, the concentration of chlamydospores tended to increase in the control group and over time in each compartment simulated. This difference could be related to the physical action (mechanical milling) of the oral cavity, the chemical or microbiological action (changes in pH, enzymes, and rumen fluid) of the other three steps of the methodology which alter the release profile of chlamydospores to the medium. These results are different from those obtained by Ojeda-Robertos et al. (2009) because *D. flagrans* was subjected to an in vitro ruminal digestion (12 h and 24 h) followed by abomasal digestion (4 h), a reduction of chlamydospores close to 36% was obtained. This difference could be related to the methodology implemented in each digestibility test, the fungal biomass employed, the strain of *D. flagrans,* and the method for its production and conservation. According to Fitz Aranda et al. (2015) the conservation method could affect the cells and therefore the nematode predatory ability of the fungus.

On the other hand, the gradual increase in the concentration of broken chlamydospores after the sequential exposure to the in vitro methodology indicates that the milling process, implemented to simulate the oral cavity, increased the surface area of the fungi exposed to the physicochemical and microbiological conditions of the subsequent segments evaluated. It is important to mention that the damage caused to these chlamydospores depends on the exposure time since in the long-exposure assay the percentage of broken chlamydospores was higher (6.87%) than that observed in the short-exposure assay (5.92%).

Chlamydospores are thick-walled resistance structures composed of different lipids, carbohydrates, and polymer molecules (Webster and Weber 2007). Melanins are amorphous polymers commonly found in fungi such as *D. flagrans* and have been associated with survival under adverse temperatures, ultraviolet radiation, or intense pressure conditions (Freitas et al. 2019). The distribution and organization of melanin in the cell wall is important for its protective effect. The mechanical damage during the mastication process could affect its structure and the deleterious effect may be evident through the subsequent exposition to the gastrointestinal cavities. Additionally, the mechanical damage could affect structures involved in the enzymatic process involved in the nematode capture mechanism (Youssar et al. 2019). As observed in this study, after subjecting *D. flagrans* to prolonged exposure times, the nematode predatory ability was lost (100%) after the sequential passage through each represented segment. Additionally, it is important to remember that digestion is carried out by the effect of enzymes such as trypsin and pancreatin, which need specific binding sites in the substrate to exercise their activity, in not broken chlamydospores, those sites may not be readily available.

In the short-exposure assay, the nematode predatory ability of *D. flagrans* was 62% which is similar to the results reported by Silveira et al. (2017) who evaluated the nematode predatory ability of two nematophagous fungi (*D. flagrans* with *Arthrobotrys robusta*) on larvae (L3) of gastrointestinal nematodes. The larvae reduction rate of the fungal combination was 53%, 72%, and 68% after 12, 24, and 48 h of transit through the gastrointestinal tract of goats respectively. Those results are similar to the obtained in our assay suggesting that these conditions may reflect what occurs in vivo.

The retention time of any feed on the digestive tract of ruminants depends of factors such as the particle size and shape, type of diet, and rumen condition (De Vega and Poppi 1997). Small particles and liquids tend to have a fast rate of outflow being quickly digested. Considering the physical characteristics of the chlamydospores of the fungi, it is more likely that they follow a rapid digestion process which is more related to a short exposure time to the gastrointestinal segments. According to Waller et al. (1994) after 4–12 h most of the chlamydospores of *Arthrobotrys oligospora* pass through the rumen of sheep. This study supports the hypothesis of a quick pass of *D. flagrans* through the gastrointestinal tract and therefore long exposure times to the rumen, abomasum and small intestine may be excessive for fungal chlamydospores. This could be related to the loss of the nematode predatory ability of *D. flagrans* after 24 h of exposition to the rumen, 3 h to the abomasum, and 24 h to the small intestine.

## Conclusions

The designed in vitro methodology showed that sequential exposure to the ovine digestive tract segments has a negative effect, especially on the nematode predatory ability of the fungi and not on the chlamydospores concentration. The effect of each segment depends on the exposure time, the longer the exposure time, the lower the nematode predatory ability of *D. flagrans*. After 51 h of exposure to the in vitro methodology, the fungi lost completely its nematode predatory ability. The number of chlamydospores with any physical alteration in the wall increased sequentially with passage through each in vitro segment of the digestive tract evaluated, this effect was more drastic in the long-exposure assay.

## Data Availability

All data generated or analysed during this study are included in this published article.
